# Embolic Protection in Complex Femoropopliteal Interventions: Safety, Efficacy and Predictors of Filter Macroembolization

**DOI:** 10.1007/s00270-020-02717-w

**Published:** 2020-12-06

**Authors:** Michael Czihal, Zeynep Findik, Christoph Bernau, Max Seidensticker, Jens Ricke, Ulrich Hoffmann, Marcus Treitl, Karla-Maria Treitl

**Affiliations:** 1 Division of Vascular Medicine, Medical Clinic and Policlinic IV, Hospital of the Ludwig-Maximilians-University, Pettenkoferstrasse 8a, 80336 Munich, Germany; 2 Clinic and Policlinic for Radiology, Hospital of the Ludwig-Maximilians-University, Munich, Germany; 3 Department of Radiology, Neuroradiology, Interventional Radiology, Berufsgenossenschaftliche Unfallklinik, Murnau, Germany

**Keywords:** Peripheral arterial disease, Percutaneous transluminal angioplasty, Atherectomy, Thrombectomy, Embolization, Filter

## Abstract

**Objectives:**

To evaluate the safety and efficacy of a filter embolic protection device (FEPD) in endovascular interventions of the femoropopliteal arteries.

**Methods:**

Patients who underwent endovascular interventions of the femoropopliteal arteries between 2008 and 2016 and in whom the SpiderFX^TM^ FEPD was applied were included in this retrospective study. Clinical and angiographic characteristics, filter macroembolization (FME), device-related complications, distal embolization, as well as the early clinical and hemodynamic outcome, were assessed. Potential risk factors for FME were evaluated by multivariate analysis.

**Results:**

A total of 244 cases were identified (203 patients, claudication 60.4%, critical limb ischaemia 39.6%, mean lesion length 13.2 ± 12.9 cm, complete occlusions in 72.7%). Balloon angioplasty ± stenting (BAP), directional atherectomy ± balloon angioplasty ± stenting (DA) and rotational thrombectomy ± balloon angioplasty ± stenting (RT) were performed in 141, 61 and 42 cases, respectively. FEPD placement and retrieval were successful in all but one case each. Permanent filter-related vessel damage was not observed. The rate of FME was 37.3% (BAP 36.2%, DA 32.8%, RT 47.7%). Risk factors for FME in the BAP- and DA-group were total occlusion, lesion length > 19 cm, visible thrombus and diabetes mellitus. The distal embolization rate despite filter protection was 4.1 % (BAP 4.9%, DA 1.6%, RT 4.8%) and was higher in cases with FME compared with those without FME (8.7% vs. 1.5%, *p *= 0.02).

**Conclusion:**

The Spider FX^TM^ device is safe and effective in capturing embolic debris during femoropopliteal interventions. A residual risk of peripheral embolization remains.

**Level of Evidence:**

III, Cohort study

## Introduction

Peripheral embolization is a well-recognized, potentially limb threatening complication of lower extremity endovascular procedures, resulting in clinically significant perfusion impairment at the cruropedal level in 1.6 to 4% of patients, with higher numbers reported in the treatment of acute thrombotic lesions (up to 24%) [1, 2]. Local pharmacological or mechanical treatment of distal embolization aims to restore distal perfusion but is associated with longer intervention times, increased radiation exposure and repeat interventions [2, 3]. Filter embolic protection devices (FEPD) have been shown to effectively capture emboli during lower extremity endovascular procedures [4–7]. However, data regarding the rational use of FEPD in lower extremity endovascular procedures are scarce and mainly limited to directional atherectomy [7, 8].

We sought to evaluate the procedural safety and efficacy of a FEPD in a large contemporary cohort of patients with acute, subacute and chronic obstructions of the femoropopliteal arteries, who either underwent balloon angioplasty ± stenting (BAP), directional atherectomy ± balloon angioplasty ± stenting (DA) or rotational thrombectomy ± balloon angioplasty ± stenting (RT). We further aimed to determine potential predictors of peri-procedural filter macroembolization (FME).

## Patients and Methods

### Study Design and Clinical Assessment

The study protocol followed the principles of the Declaration of Helsinki. Patients who underwent endovascular interventions of the femoropopliteal arteries (and additionally of the iliac inflow and the crural outflow, if required) between 2008 and 2016 were retrospectively identified. Patients aged ≥ 18 years suffering from symptomatic peripheral arterial disease Rutherford stage 2–6 were eligible for study inclusion, provided that the target lesion was successfully crossed by a guidewire.

Clinical data (symptom duration, cardiovascular comorbidities, current medication and previous revascularization procedures) were obtained from the medical records. Laboratory values as well as pre- and post-interventional hemodynamic parameters (systolic ankle pressures, ankle brachial index and segmental pulse volume recordings of the forefoot) were recorded.

### Endovascular Procedures

All procedures were performed by a single, experienced interventional radiologist (M.T.). The endovascular procedures were carried out using 6–8F sheaths (antegrade access or retrograde access with crossover-manoeuvre, as appropriate) and after intraarterial administration of 5.000IU. of heparin.

The choice of a single revascularization technique or a combination of more than one technique was at the discretion of the operator, based on symptom duration and angiographic appearance. In general, DA was performed in patients with chronic symptoms resulting from arteriosclerotic lesions (mainly stenoses), whereas RT was applied in (sub-)acute occlusions of native arteries and prosthetic bypass grafts with high clinical suspicion of thrombotic material. BAP was used for treatment of both chronic arteriosclerotic lesions and (sub-)acute occlusions. DA and RT procedures all were performed under mandatory filter protection. In BAP procedures, utilization of the FEPD was indicated when, based on clinical information (e.g. symptom duration, previous revascularization) and angiographic appearance (e.g. visible thrombus, flush occlusion of a native artery or a bypass graft), an increased periinterventional embolization risk was anticipated.

Standard balloon catheters were used for BAP. DA was carried out with devices of the Hawk^TM^-family (Medtronic Vascular, Santa Rosa, CA, USA). RT was carried out with Rotarex^TM^-catheter systems (Straub Medical AG, Wangs, Switzerland). DA and RT were followed by adjunctive BAP, if necessary. If required (residual stenosis > 30%, flow limiting dissection), adjunctive stenting with self-expanding nitinol stents (open-cell design) of various manufacturers was performed. Stent implantation was preceded by lesion preparation in all cases. In some acute and subacute thromboembolic occlusions, aspiration thrombectomy and/or local thrombolysis was applied in addition to the above-mentioned recanalization procedures.

Following lesion crossing with a 0.018 V-18TM ControlWire (Boston Scientific, Malborough, MA, USA) and prior to revascularization, the SpiderFX^TM^ (EV3, Mansfield, MA, USA) was inserted. The filter basket (size 3 to 7 mm, chosen according to the diameter of the vessel lumen as determined by digital subtraction angiography) was deployed at least 2 cm below the target lesion either in a popliteal or, in case of single vessel crural run-off, in a below-the-knee vessel segment. After release of the FEPD, digital subtraction angiography images were obtained in two projections in order to confirm adequate contact of the FEPD to the vessel wall. After completed revascularization, the FEPD was retrieved using the manufacturer-provided capturing catheter. Final completion angiography of the treated lower extremity was performed in order to document technical success and to assess distal run-off with regard to peripheral embolization.

Post-interventional medical treatment consisted of dual antiplatelet therapy with aspirin and clopidogrel for three to six months, with the loading dose of clopidogrel (300 mg) given immediately after the procedure. In patients with an indication for oral anticoagulation (e.g. infrainguinal venous bypass, atrial fibrillation), aspirin was combined with oral anticoagulants at therapeutic doses instead of clopidogrel.

### Evaluation of Procedural Parameters

An experienced interventional radiologist (M.T.) reviewed the digital subtraction angiography sequences. Lesion characteristics (target vessel diameter, lesion length, grade of calcification, visible thrombus, number of patent distal run-off vessels in the index angiography and the final angiography) were analysed according to a predefined protocol. Peri-procedural FEPD-related complications were recorded, including problems with filter management, filter-induced local vasospasm and permanent filter-related permanent vessel damage.

**Fig. 1:**
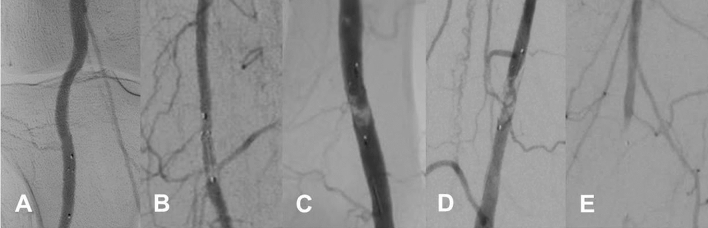
Angiographic examples of different grades of embolic debris captured in the filter basket. 0, no visible material (**A**); 1, sludge with partial occlusion of filter meshes or single debris particles (**B**); 2, macro-debris filling less than one-third of the filter (**C**); 3, macro-debris filling more than one-third of the filter (**D**); 4, completely filled filter basket (**E**)

Debris within the filter was classified using a modified semiquantitative scoring system based on the appearance of the filter basket during final magnified angiography, as follows: (0) no visible material; (1) sludge with partial occlusion of filter meshes or single debris particles (2) macro-debris filling less than one-third of the filter; (3) macro-debris filling more than one-third of the filter; (4) completely filled filter basket (Fig. 1) [9]. Debris within the filter ≥ grade 2 was considered to be FME of potential clinical relevance.

### Outcome Parameters

The following outcome parameters were assessed:

- Successful filter management: placement and retrieval of the FEPD without complications.

- Permanent filter-related damage: dissection, stenosis/occlusion, perforation considered to be caused by the filter basket/filter wire.

- Technically successful revascularization: restoration of a regular blood flow at the lesion level with residual stenosis < 30% and patent crural run-off (at least one artery).

- Hemodynamic improvement: increase in ankle brachial index > 0.15 and/or significant improvement of forefoot pulse volume recordings by semiquantitative assessment [10].

- Peripheral embolization: occurrence of a new angiographic filling defect in a tibial, fibular or pedal artery observed during the procedure or at final completion angiography.

- FME of potential clinical significance: debris within the filter ≥ grade 2 was considered to be FME of potential clinical relevance, given its potential to occlude a tibial or fibular artery.

### Statistical Analysis

Statistical analysis was performed with the R software for statistical computing (R Development Core Team, Vienna, Austria). Results for categorical variables are presented as absolute numbers/percentages, and continuous variables are displayed as mean ± standard deviation. For univariate analysis, Fisher’s exact test and Wilcoxon’s rank sum test were applied. Correction for multiple testing was done using the Bonferroni method. To identify independent predictors of FME, multiple logistic regression models were calculated. Finally, decision tree analysis was performed. Two-sided *p* values < 0.05 were considered statistically significant.

## Results

### Cohort Characteristics

The main clinical and angiographic characteristics of the 244 included cases (203 patients) are summarized in Table 1. Briefly, the mean age of the cohort was 71.4 ± 10.9 years, and 46.1% were women. Indication for intervention was claudication (Rutherford categories 2 and 3) in 60.4% and critical limb ischaemia (Rutherford categories 4–6) in 39.6%. Acute symptoms lasting less than 14 days and subacute or chronic symptoms (> 14 days) were present in roughly half of the patients, respectively (53.5 vs. 46.5%). Mean lesion length was 13.2 ± 12.9 cm, and total occlusions were present in 72.7% of cases. Occlusions of femoropopliteal or femoro-distal bypasses accounted for 15.2% of cases. A three-, two- and one-vessel run-off were present in 18.9%, 36.9% and 40.1% of cases, respectively. Ten patients (4.1%) had no patent crural artery.

**Table 1:** Comparison of clinical and lesion characteristics between patients treated by balloon angioplasty ± stenting (BAP), directional atherectomy ± balloon angioplasty ± stenting (DA) and rotational thrombectomy ± balloon angioplasty ± stenting (RT)

Variable	BAP *n* = 141	DA *n* = 61	RT *n* = 42
Age, years	71.7 ± 10.9	71.6 ± 9.4	70.3 ± 12.9
Male sex (%)	56.3	60.7	35.7
Arterial hypertension, %	78.9	93.3	78.0
Diabetes mellitus, %	23.9	42.6	14.3
Current or former smoking, %	57.1	65.6	53.7
Dyslipidemia, %	63.4	73.3	47.6
Coronary heart disease, %	30.3	23.0	35.7
Cerebrovascular disease, %	16.9	23.0	19.0
Statin treatment, %	54.3	58.3	57.1
Antiplatelet therapy, %	69.7	80.3	78.6
Dual antiplatelet therapy, %	12.7	8.2	19.0
Symptom duration, days	61 ± 136	104 ± 162	16 ± 32
Acute symptoms < 14 days, %	56.6	29.8	71.4
Critical limb ischaemia, %	42.2	31.2	42.9
ABI	0.54 ± 0.39	0.66 ± 0.29	0.28 ± 0.25
Total occlusions, %	78.9	39.3	100
Recurrent lesions, %	37.3	54.1	71.4
Lesion length, cm	13.5 ± 12.6	11.2 ± 11.4	15 ± 15.6
Visible thrombus, %	22.7	19.7	33.3
Moderate/severe calcification, %	31.2	37.7	45.2
Distal run-off ≤ 1 vessel	47.6	36.1	45.3
Adjunctive stenting, %	66.2	19.7	38.1
FME, %	36.2	32.8	47.7
Distal embolization, %	4.9	1.6	4.8

Continuous data are given as means ± standard deviation; categorical data are given as counts (percentage). FME, filter macroembolization; ABI, ankle brachial index.

DA and RT were performed in 61 (25%) and 42 (17.2%) patients, respectively. The remaining 141 patients underwent BAP without any debulking procedure. Adjunctive aspiration thrombectomy and/or local thrombolysis was performed in 28.3 and 17.6% of cases, respectively. Additional endovascular treatment of iliac artery inflow or crural artery outflow was performed in 2.0 and 31.1% of cases.

Of note, the rate of patients with acute symptoms was higher in the groups treated by RT (71.4%) and BAP (55.6%) compared with DA (29.8%). Correspondingly, the rate of patients with total occlusions was substantially higher in these groups (RT 40.5% vs. BAP 55.3% vs. DA 3.3%). Aspiration thrombectomy and/or thrombolysis was performed more frequently in the RT-group (40.5%) and BAP-group (55.3%) compared with the DA-group (3.3%).

### FEPD-Related Outcome

Deployment of the FEPD was successful in all but one case. This patient was consequently treated with unprotected RT which was complicated by peripheral embolization. In another single case, retrieval of the FEPD was not possible due to an overfilled filter, necessitating FEPD removal in conjunction with the introducing sheath without further complications. Thus, the rate of successful filter management was 99.2%.

Permanent filter-related vessel damage was not observed. However, vasospasm at the filter site requiring intraarterial spasmolysis with nitroglycerine occurred in 11 cases (4.5%), including seven cases with filters at the crural level and four cases with filters at the popliteal level.

### Procedural and Clinical Outcome

Technically successful revascularization was achieved in 96.7% of cases. The corresponding rate of post-procedural hemodynamic improvement was 93.0%. Revascularization failed in eight cases (3.3%), including two patients with primary technical failure and one patient in whom perforation of the popliteal artery following RT required urgent surgical revascularization. Five patients experienced early re-occlusions of the treated vessel segment within 24 hours of the procedure.

Despite filter protection, peripheral embolization distal to the FEDP occurred in ten cases (4.1%; BAP *n *= 7; RT *n *= 2; DA *n *= 1), including nine total occlusions. The mean symptom duration in these patients was 80 days (range 4–365 days; four cases with acute symptoms <  14 days), and mean lesion length was 17.2 ± 16.4 cm (range 8–42 cm). In five of these cases, aspiration thrombectomy was attempted prior to filter placement. In eight of the ten cases with peripheral embolization distal to the FEDP, macroemboli were also found in the filter basket. Treatment consisting of aspiration thrombectomy (*n *= 3) and/or thrombolysis (*n *= 8) was successful in nine of the ten cases. After the procedure, hemodynamic improvement was documented in all ten cases. Within a mean follow-up time of 2.8 + 2.9 years, freedom from target lesion revascularization was documented in only two of these ten cases. Eight re-occlusions occurred after a mean of 0.9 ± 1.4 years, prompting revascularization procedures in six patients (two of whom required bypass surgery). One patient subsequently underwent lower limb amputation.

### Risk Factors for FME

Overall, peri-procedural FME considered to be of potential clinical significance (≥ grade 2) occurred in 37.3% of cases. FME was more common in patients who underwent RT compared with DA and BAP (47.7% vs. 36.2% vs. 32.8%). Peripheral embolization distal to the FEPD was documented in 4.1% of all patients and was significantly more common in cases with FME compared with those without FME (8.7% vs. 1.5%, *p* = 0.02).

**Fig. 2:**
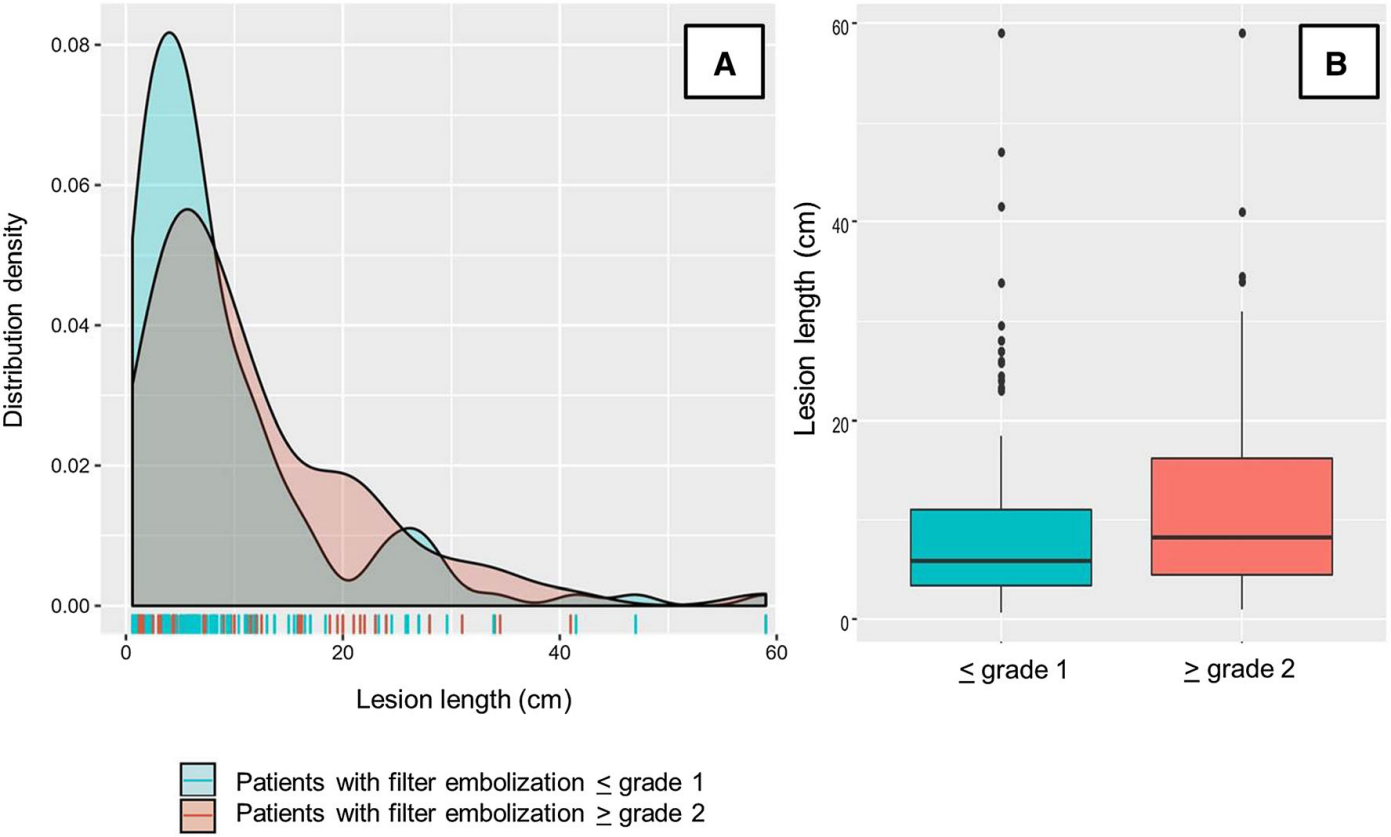
Overlapping distribution of cases with and without FME in relation to lesion length (**A**). Comparison of mean (± standard deviation) lesion length between patients with and without FME (**B**)

Given the excessive risk of FME associated with RT, further analysis evaluating risk factors for peri-procedural embolism was restricted to the groups with DA and BAP only. Results of the univariate analysis are listed in Table 2. There was a trend towards a higher FME rate in cases with critical limb ischaemia compared with cases with claudication, but a significant difference between patients with and without FME was only found for mean lesion length (≥ grade 2: 15.5 ± 13 cm vs. ≤ grade 1: 11.3 ± 11.6 cm, *p *< 0.01). Notably, there was a remarkable overlap between lesion lengths in patients with and without FME (Fig. 2). A marked increase in the rate of FME was seen in lesions exceeding a length of 19 cm when compared with lesions shorter than 19 cm (FME rate 51.6 vs. 30.8%, *p* = 0.04).

**Table 2:** Comparison of clinical and lesion characteristics of patients with and without filter macroembolism (analysis limited to patients treated by BAP and DA; patients treated by RT were excluded)

Variable	No FME *n* = 133	FME *n* = 69	*p* value
Age, years	71.5 ± 9.8	71.7 ± 11.5	0.88
Male sex (%)	58.5	56.3	0.77
Arterial hypertension, %	80.8	87.3	0.33
Diabetes mellitus, %	26.0	36.6	0.15
Current or former smoking, %	59.5	70.5	0.69
Dyslipidemia, %	64.6	70.4	0.44
Coronary heart disease, %	27.5	29.6	0.75
Cerebrovascular disease, %	19.1	18.3	1.0
Statin treatment, %	51.2	62.9	0.14
Antiplatelet therapy, %	63.4	56.3	0.38
Dual antiplatelet therapy, %	11.5	11.0	1.0
Symptom duration, days	69 ± 151	79 ± 135	0.50
Acute symptoms < 14 days, %	51	45.5	0.62
Critical limb ischaemia, %	35.1	45	0.07
ABI	0.6 ± 0.36	0.53 ± 0.37	0.16
Total occlusions, %	69.5	62.0	0.35
Recurrent lesions, %	41.2	45.1	0.35
Lesion length, cm	11.3 ± 11.6	15.5 ± 13	< 0.01
Visible thrombus, %	18.3	28.2	0.11
Moderate/severe calcification, %	29	40.9	0.23
Distal run-off ≤ 1 vessel	42	47.8	0.41
Adjunctive stenting, %	51.1	53.5	0.77
Distal embolization, %	1.5	8.7	0.02

Continuous data are given as means ± standard deviation; categorical data are given as counts (percentage). FME, filter macroembolization; ABI, ankle brachial index.

Logistic regression analysis favoured a model including the following variables: total occlusion, lesion length > 19 cm, visible thrombus, DA procedure and chronic antiplatelet therapy. However, comparison between patients with and without chronic antiplatelet therapy showed significantly worse patient- and lesion-based risk profiles in the 73.9% of patients who were under chronic (> 14 days of treatment) antiplatelet therapy prior to the procedure (significantly higher prevalence of coronary artery disease and current smoking, longer mean lesion length, higher rate of recurrent lesions; data not shown). Therefore, the variable antiplatelet therapy was excluded from the final model, which included the following parameters: total occlusion, lesion length > 19 cm, visible thrombus, DA procedure and diabetes mellitus. There was a significant interaction between visible thrombus and diabetes mellitus, leading to a marked risk increase for FME (83%) for this small subset of patients (19 patients, 7.9% of the overall study population) with both factors.

**Fig. 3:**
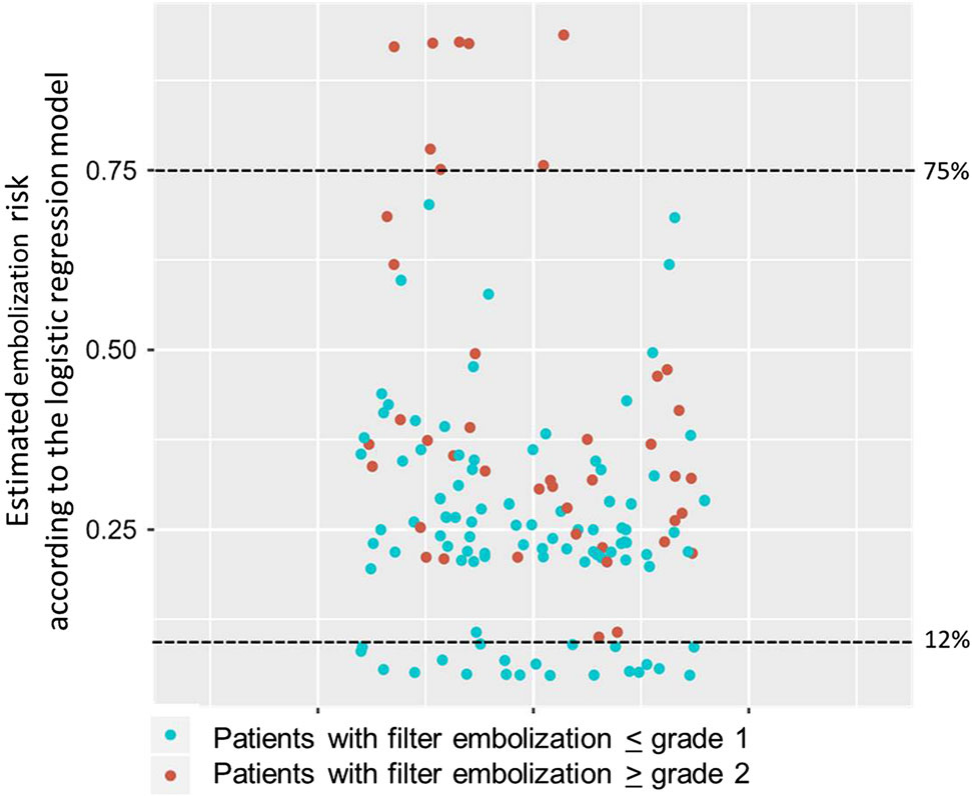
Prediction of cases without (≤ grade 1) and with (≥ grade 2) FME by the final logistic regression model in the subset of patients treated with BAP (*n* = 141)

**Fig. 4:**
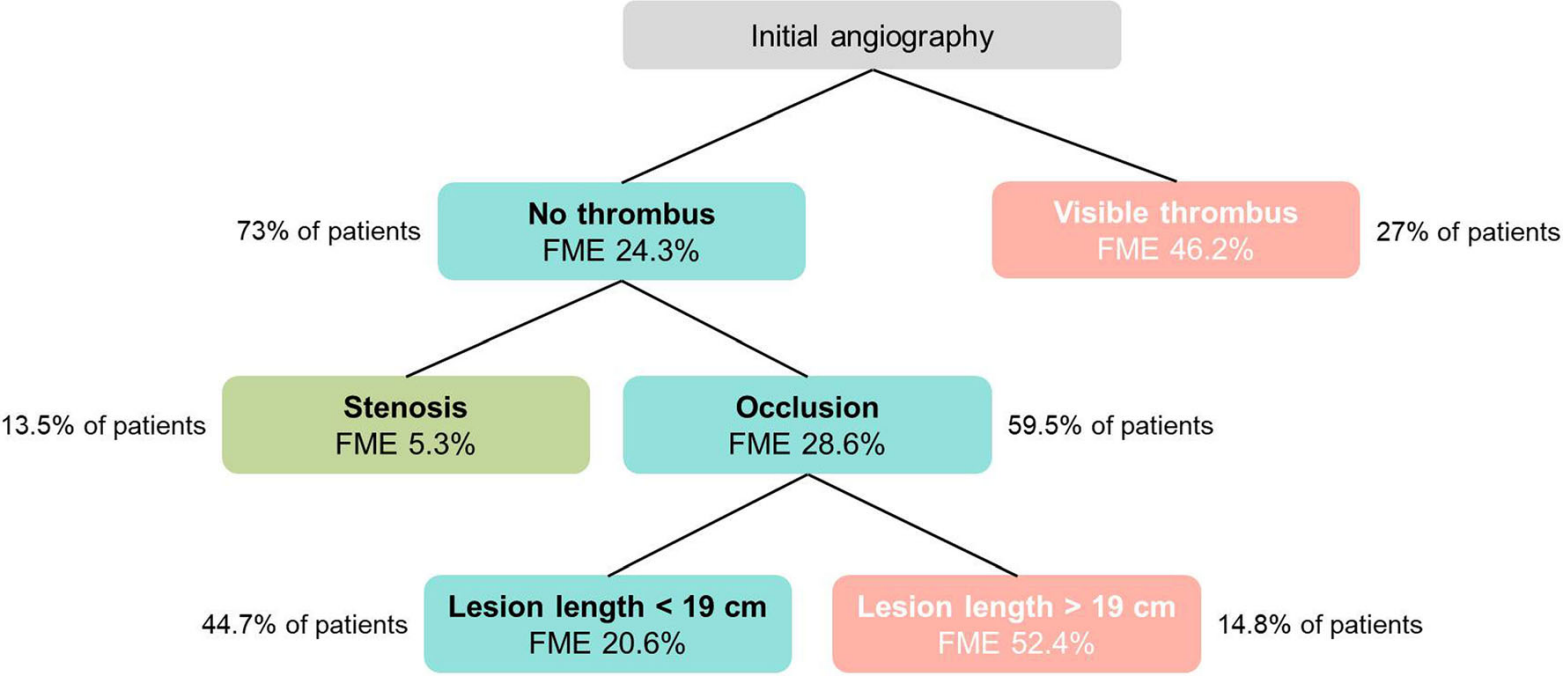
Decision tree analysis for the subset of patients treated with BAP (*n* = 141), based on variables derived from the final logistic regression model. FME risk is visualized by different colours (red: high risk of FME; turquoise: low to intermediate risk; green: very low risk)

The model showed a substantial discriminatory value for patients with high, intermediate and low embolization risk. In the group of patients treated with BAP, the model allowed exact identification of a low-risk group (embolization risk < 12%) and a high-risk group (embolization risk > 75%) (Fig. 3). These findings were underscored by decision tree analysis which discriminated a subset of cases at very low risk (stenosis without visible thrombus) from subgroups with low to moderate risk (occlusions < 19 cm without visible thrombus) and high risk (occlusions > 19 cm, lesions with visible thrombus) of FME (Fig. 4). 

## Discussion

FEPD appeared to be safe and effective in capturing embolic debris during complex endovascular interventions of the femoropopliteal arteries (overall rate of FME of 37.6%). Distal embolization despite filter protection occurred in 4.1% of cases. The comparatively high rates of both FME and distal embolization are mainly attributable to the cohort characteristics, with large proportion of cases presenting with acute symptoms and/or complete occlusions particularly in the groups treated by BAP or RT (more than 50% of the cases).

RT of (sub-)acute femoropopliteal occlusions was associated with a rate of FME approaching 50% and a distal embolization rate of 4.8%. Data on the risk of FME during mechanical thrombectomy procedures are scarce. Not surprisingly, Karnabatidis et al. identified mechanical thrombectomy procedures as independent risk factors of FME [11]. In older studies, distal embolization rates between 25 and 56% were reported with various mechanical thrombectomy procedures [12]. Recently published, large cohort studies showed distal embolization rates of 5.5 and 12.7% with RT [13, 14]. Altogether, these data support the routine use of FEPD when performing RT of acute and subacute femoropopliteal occlusions.

In previous DA series, the reported rates of FME varied widely, ranging from 9 to 91% [4, 6–8]. In the Definitive LE study, including almost 800 participants with chronic limb ischaemia, distal protection was at the discretion of the operator. Consequently, FEPD was used in only 20% of patients during DA, and the distal embolization rate was 3.8% [15]. In the Definitive Ca^++^ study, distal embolization occurred at a lower rate in three out of 111 patients with chronic limb ischaemia (2.3% of the study cohort) who underwent filter-protected DA [6]. In our study, the distal embolization rate during filter-protected DA was 1.6%. Based on a large series (*n* = 508) with patients who underwent femoropopliteal DA with utilization of a FEPD, Krishnan et al. were the first to propose an algorithm for the rational use of FEPD in DA procedures [8]. Their model included chronic total occlusions, in-stent-restenosis, thrombotic lesions, lesions > 140 mm, calcified lesions > 40 mm and the number of run-off vessels.

The role of FEPD in the setting of BAP of femoropopliteal lesions is less well defined. In large series without filter protection, the rates of clinical significant distal embolization were between 1.6 and 2.4% [2, 3]. In the subset of cases treated by FEPD-protected BAP in our series, the rate of significant distal embolization was even higher (4.9%). However, this does not implicate that FEPD paradoxically increases the risk of distal embolization but rather reflects the characteristics of our preselected cohort with a very high proportion of patients with (sub-)acute and long occlusions. Correspondingly, in our series, every third patient exhibited FME after BAP. Müller-Hülsbeck et al. found FME (filter basket filled > 1/3) in three of 30 stenotic lesions of the femoropopliteal arteries treated with BAP [9]. In the PROTECT-registry, FME occurred in 27.6% of subjects treated with BAP [4]. Mendes et al. observed moderate to severe debris burden in 45% of filter baskets during 87 femoropopliteal interventions (98% BAP) [2]. By contrast, Spiliopolus et al. in a recent study found microdebris in all filters but no evidence of FME in 40 patients who underwent subintimal recanalization of chronic femoropopliteal occlusions [5].

In our analysis, thrombotic and long lesions, as well as chronic occlusions, appeared to be associated with a particularly increased risk of FME also in the subgroup of patients with BAP, whereas other parameters such as symptom duration, lesion calcification and number of run-off vessels were not. Chronic total occlusions, lesion length, as well as thrombus burden, were positively correlated with the amount of captured particles in the series by Karnabitides et al. [11]. According to the available data, the use of FEPD during BAP in lesions with visible thrombus burden, as well as in patients with long occlusions, may be advisable.

In patients with compromised crural run-off, FEPD may be used even more liberally to avoid clinically relevant outflow deterioration [13]. The potential role of a “microcirculatory injury”, well recognized in percutaneous coronary interventions in acute myocardial infarction, is insufficiently understood in the context of femoropopliteal interventions [5, 16]. However, the ubiquitous phenomenon of clinically silent microembolization may be of importance particularly in repeated procedures [5]. Given their excellent safety profile, as documented for the SpiderFX^TM^ in our study, it is worth discussing the utilization of FEPD at least in subjects considered to be at high risk for distal embolization or with a predicted poor outcome in case of distal embolization.

However, the level of evidence regarding the rational use of FEPD in peripheral interventions remains low and the additional costs must be taken into account. A huge discrepancy exists between high rates of FME and the remarkable lower rate of clinically significant distal embolism in unprotected procedures. Noteworthy, in our study, the rate of distal embolization was significantly higher in cases with FME than in those without (8.7 vs. 1.5%). A consensus definition of how to define “clinically significant” FME does not exist. While some studies provided no clear definitions of FME [2, 6, 8] and some studies graded FME by particle diameter [4, 7], we applied a modified semiquantitative evaluation of the debris load in filter baskets based on final angiography before filter retrieval [9]. It must be noted that our definition is arbitrary and not evidence-based. Thus, it still remains to be evaluated which mode of debris analysis is preferable within clinical studies.

Our retrospective study has some limitations, mainly the inhomogeneous patient cohort, performance of endovascular procedures by a single operator who was involved in analysis of procedural parameters and the lack of a core laboratory analysis. The comparatively high rates of both FME and distal embolization are mainly attributable to an obvious selection bias, as stated above.

As most studies with large patient numbers relied on retrospective data with the inherent risk of bias [2, 8], and prospective studies with mandatory FEPD use were mainly performed in the setting of DA [6, 7], evaluation of the potential clinical benefit and of the cost-effectiveness of FEPD in the setting of a properly designed, prospective, randomized, controlled study is warranted. Risk algorithms as stated above could be helpful in enriching the study population with patients carrying a high peri-procedural embolization risk.

In summary, our study showed an excellent safety profile and the ability of the SpiderFX^TM^ to effectively capture macro-debris during different types of femoropopliteal interventions. A residual risk of distal embolization remains, and the discrepancy between high rates of FME and much lower rates of distal embolization in unprotected cases represents an unsolved problem. Our results may inform future research aiming at determining the clinical benefit of FEPD in certain clinical scenarios of femoropopliteal interventions.
